# Congenital hypothyroidism and thyroid dysgenesis: importance of newborn bloodspot screening in Ireland

**DOI:** 10.1007/s11845-026-04280-8

**Published:** 2026-02-14

**Authors:** Alison McHugh, E. O’Connell, S. Hoare, C. Heffernan, E. Carolan

**Affiliations:** 1 Department of Paediatric Otolaryngology, CHI at Temple St, Dublin, Ireland; 2 Department of Endocrinology, CHI at Temple St, Dublin, Ireland; 3 Department of Radiology, CHI at Temple St, Dublin, Ireland; 4 Royal College of Surgeons, 123 St Stephen’s Green, Dublin, Ireland

**Keywords:** Congenital, Thyroid, Hypothyroid, Dysgenesis, Hemiagenesis, Sublingual

## Abstract

**Introduction:**

Congenital hypothyroidism, which is screened for on the national newborn bloodspot screening test, has potentially devastating developmental consequences for a child if left untreated. The disease may be otherwise innocuous in a newborn with symptoms including fatigue, poor feeding and constipation. Outside of the need for supplemental levothyroxine, an ectopic thyroid gland and hemi-agenesis bear no additional risk to the individual once identified and treated.

**Case report:**

Congenital hypothyroidism was detected in an otherwise asymptomatic neonate on the newborn heel-prick screening test. Confirmatory serum analyses of both the infant and mother were performed. Thyroid scintigraphy and thyroid ultrasound confirmed the absence of an orthotopic thyroid gland, with a solitary ectopic thyroid lobe detected sublingually. The infant was started on supplemental levothyroxine and titrated until serum thyroid levels normalised. Developmental milestones and serial parameters, including weight, height, and head circumference, were normal.

**Discussion:**

Congenital hypothyroidism, which is screened for on the national newborn bloodspot screening test, has potentially devastating developmental consequences for a child if left untreated. The disease may be otherwise innocuous in a newborn with symptoms including fatigue, poor feeding and constipation. Outside of the need for supplemental levothyroxine, an ectopic thyroid gland and hemi-agenesis bear no additional risk to the individual once identified and treated.

## Introduction

Congenital hypothyroidism (CHT), in the absence of screening, can result in significant neurodevelopmental delay and intellectual disability [1]. Congenital hypothyroidism is screened for in the Republic of Ireland on the Newborn Bloodspot Screening test (or Guthrie test) by collecting a heel-prick blood sample between 72 and 120 h after birth [2]. 

## Case based review

### Presentation

A neonate was born to a 32 year old G1P1 woman, age 32, at 38 + 6 weeks gestation via elective caesarean section for unstable lie. Due to a spontaneous pneumothorax on day of life (DOL) 0, a chest drain was inserted and the infant was intubated. Her newborn heel-prick screening test (Guthrie test) was performed DOL6 following extubation and return to full oral feeds.

### Diagnosis

On the Guthrie test, she was found to have an elevated thyroid-stimulating hormone (TSH) 98 miu/l. Confirmatory serum thyroid function tests (TFTs) performed on DOL7 showed an elevated TSH 210 miu/l and thyroxine (T4) 17.2 pmol/l. The infant was clinically well, breastfeeding and gaining weight. She passed her newborn hearing screening test. Levothyroxine replacement therapy was commenced at 50 micrograms per oral (PO) daily and she was referred to paediatric endocrinology.

**Fig. 1:**
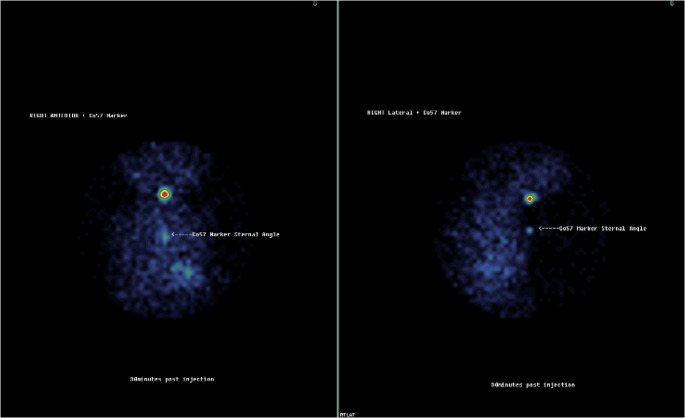
Nuclear medicine study (8.5 MBq Technetium 99 m uptake scan) on right lateral and anterior view showing uptake in the sublingual area with no uptake in the neck

**Fig. 2:**
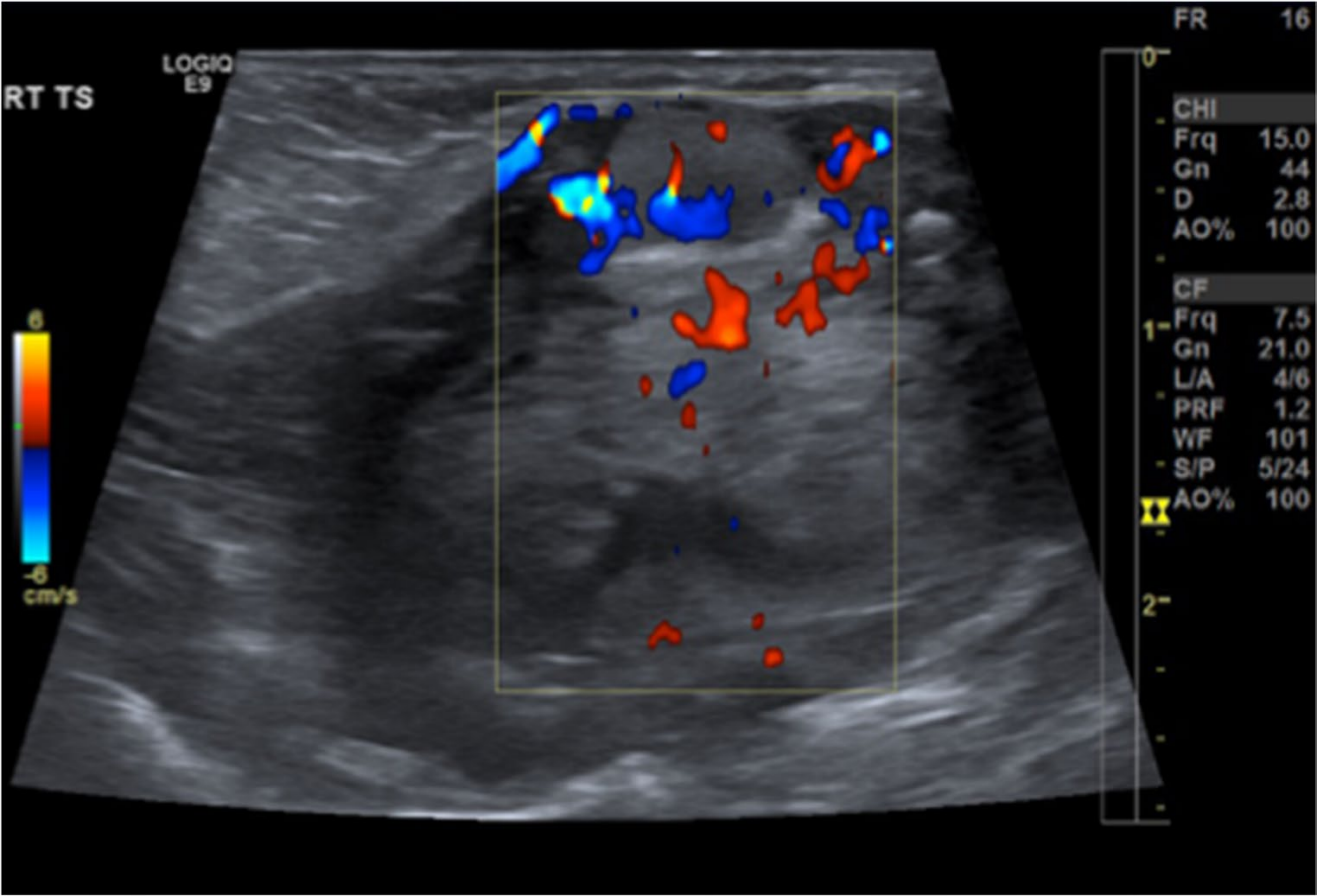
Ultrasound neck with Doppler showing a 6.5 mm x 5.6 mm nodule of thyroid tissue high in the neck above the level of the hyoid cartilage with vascularity on Doppler

Maternal thyroid peroxidase (TPO) antibodies and TFTs were normal. Thyroid scintigraphy was performed following intravenous injection of 99-m technetium on standard anterior and lateral views, showing an uptake in the sublingual area. There was no uptake in the neck. Thyroid ultrasound confirmed a sub-centimetre nodule of ectopic thyroid tissue sublingually, diagnosing a sublingual thyroid with hemiagenesis (Figs. 1 and 2).

### Treatment

Serum TFTs were monitored six weekly, normalising within 4 weeks with oral levothyroxine dose titration to 37.5 mcg daily.

The baby is developing well and meeting milestones. She is stable on her centiles (91st ) and maintains good sleep patterns and a healthy appetite.

## Discussion

CHT, a failure of thyroxine production, may be secondary to thyroid agenesis (absent thyroid), dysgenesis (abnormal thyroid growth) or dyshormonogenesis (abnormal thyroid hormone synthesis). While dyshormonogenesis can be transient or resolve within the first few years of life, thyroid dysgenesis usually requires lifelong levothyroxine replacement [3]. The underlying cause of CHT can be distinguished with thyroid scintigraphy and ultrasound.

Thyroid dysgenesis occurs due to abnormal thyroid formation or descent during embryogenesis and can include thyroid ectopy, which is almost invariably sporadic [4, 5]. The prevalence of thyroid dysgenesis and thyroid ectopy is not known, as cases may be asymptomatic. Thyroid hemiagenesis (single thyroid lobe), in particular, is often asymptomatic as a single thyroid lobe usually functions adequately in isolation. It is often only detected when a lesion appears in a solitary functioning lobe [6]. 

CHT has been screened for on the Newborn Bloodspot Screening Programme since 1979. Ireland has a higher incidence of congenital hypothyroidism (1 in 2,300) compared to the worldwide incidence (1 in 3500) [3]. Despite a consistent screening cutoff, the incidence has increased significantly over the past four decades, hypothesised to be due to increased survival of preterm infants and increased heterogeneity of the Irish population [2]. Infants with CHT are often asymptomatic so newborn screening is vital to detect this condition before long term deleterious effects occur. Similar established newborn screening programmes are not universally available worldwide with approximately 71% of infants being born into a geographical area, often in a developing country, where lack of same prohibits early diagnosis, treatment and poses a significant global public health challenge [7]. 

Compared to other conditions on the Guthrie test, false positives are more common for CHT. TSH levels may be transiently raised after birth. Antiseptic preparations containing iodine may be absorbed through neonates’ skin, resulting in transiently raised TSH levels. This infant required a chest drain insertion and as such this was a consideration for initial elevated TSH level reading as a potential false positive [3]. 

Where TSH is > 15mU/L on heel prick screening, the neonate is referred to paediatric endocrinology. The infant’s and the mother’s bloods are taken, measuring TFTs and TPO antibodies. Ideally, formal bloods and imaging will precede the commencement of levothyroxine. If TSH is very elevated or the baby is approaching ten days of age, levothyroxine is started before imaging to avoid treatment delays [3]. As serum levels were very high in this infant, she was commenced on levothyroxine before review.

CHT is investigated radiologically with thyroid scintigraphy using a radiotracer, Technetium 99 m, to detect functioning thyroid tissue. A thyroid ultrasound further assesses for the presence of normal orthotopic thyroid tissue [2]. 

Infants with CHT are placed on titrating levothyroxine with regular serum TFTs. Ectopic thyroid glands, similar to orthotopic thyroid glands, may have thyroid pathologies, including cysts, goitre, adenoma, thyroiditis, hypothyroidism, and hyperthyroidism. Malignant transformation in ectopic thyroid glands is rare [4]. 

## Conclusion

Congenital hypothyroidism carries the risk of significant developmental delay. Early intervention is easy, cost-effective and most importantly allows avoidance of the detrimental impacts this otherwise innocuous anomaly would have without early diagnosis and screening.
